# Advancing Diversity in Microbiology: A 55-Year Retrospective Analysis

**DOI:** 10.7759/cureus.52528

**Published:** 2024-01-18

**Authors:** Ameneh Marzbanrad, Farhad Niaghi, Sabeen Tiwana, Javed Siddiqi, Jeffrey Ding, Imrana Tanvir, Faisal Khosa

**Affiliations:** 1 Nutrition, McGill University, Montreal, CAN; 2 Emergency Radiology, University of British Columbia, Vancouver, CAN; 3 Dentistry, University of British Columbia, Vancouver, CAN; 4 Neurosurgery, Desert Regional Medical Center, Palm Springs, USA; 5 Neurosurgery, Riverside University Health System Medical Center, Moreno Valley, USA; 6 Neurosurgery, Arrowhead Regional Medical Center, Colton, USA; 7 Neurosurgery, California University of Science and Medicine, Colton, USA; 8 Medicine, University of British Columbia, Vancouver, CAN; 9 Pathology, King Abdulaziz University Faculty of Medicine, Jeddah, SAU; 10 Radiology, Vancouver General Hospital, Vancouver, CAN

**Keywords:** microbiology, leadership, minority groups, sexism, affirmative action

## Abstract

Background

For over 50 years, affirmative action helped advance equity, diversity and inclusion (EDI) in educational institutions in the United States (U.S.). However, the recent U.S. Supreme Court decision to end affirmative action in college admissions threatens the progress toward EDI.

Objective

This study aimed to assess the progress in promoting gender and racial diversity within the discipline of microbiology over a 55-year period. We sought to analyze the representation of women and minority groups in faculty ranks, tenure positions, and leadership to identify disparities and trends and determine who will likely be impacted most with the end of affirmative action.

Materials and methods

This longitudinal retrospective study utilized publicly available and non-identifiable Association of American Medical Colleges (AAMC) data on full-time microbiology faculty from 1967 to 2021. Faculty members were categorized based on academic ranks and tenure status, while gender and racial data were also considered.

Results

The analysis revealed a consistent dominance of white faculty, with over 60% representation across all academic ranks throughout the study period. The Asian and female faculty representation decreased in senior academic ranks. We observed a positive trend in the annual increase of women in faculty positions, academic ranks, chairs, and tenure positions. Furthermore, Asian faculty demonstrated the most robust surge in representation. However, disparities persisted for black, Hispanic, and Native American faculty members, reflecting broader challenges in their representation.

Discussion

Although efforts to enhance diversity within microbiology have yielded positive results, underrepresented minority groups still face obstacles in attaining leadership positions and senior academic ranks. The diminishing proportion of women at higher academic ranks raises concerns about potential attrition or lack of promotion opportunities. The end of affirmative action poses a risk of perpetuating this trend, leading to a decline in diversity among microbiology faculty.

## Introduction

The concern surrounding the representation of women and underrepresented minorities (URMs) in professional and scientific roles has been omnipresent. Efforts for achieving gender and race parity did bear fruit and resulted in improved representation. On June 29, 2023, the U.S. Supreme Court ended affirmative action in college admissions. This program, in place for decades, was designed to increase access to U.S. universities for underrepresented populations, specifically women and individuals from minority communities [1]. This Supreme Court ruling came at a time when there was substantial growth of racial and ethnic minorities, according to the 2020 U.S. Census Report [2].

Statistical data from 2015 reveal a notable disparity between the percentage of women in the college-educated U.S. workforce in science and engineering and their representation in actual science, technology, engineering, and mathematics (STEM) occupations [3,4]. The 2017 National Institutes of Health (NIH) report highlights gender disparities in science, revealing that among 16 NIH directors, only one was a woman. In the top 10 U.S. research institutes, the percentage of tenured women professors ranged from 20% to 26%. In the NIH intramural research program, women constituted 37% of tenure-track positions, but only 21% attained tenured status, with women of colour holding just 5% of tenured positions. Similar trends are observed in U.S. Ph.D. programs, where the percentage of female applicants increased from 38% in 2000-2001 to 57% in 2016-2017, yet women only represented 30% of all faculty in these programs [5].

These figures highlight the continued underrepresentation of women, indicating a need for further investigation into the factors influencing such trends. Moreover, the progression of women through academic ranks in life sciences demonstrates a significant gap between Ph.D. graduates and those occupying postdoctoral and tenure-track positions, signalling potential barriers impeding the advancement of women in scientific careers [5,6]. Gender bias is proposed as a contributing factor, impacting all women and having a heightened effect on those with intersecting identities subject to discrimination, such as race and ethnicity. The pervasive nature of gender biases is not limited to academia; they are deeply ingrained in societies and manifest early in life, influencing young girls' career aspirations and lifelong educational achievements [5].

The analysis of population trends indicates a significant surge in the population of URMs within the U.S. Projections suggest that by 2044, these URMs are expected to make up more than half of the U.S. population [7-9]. Despite this evolving demographic landscape, disparities based on race and gender continue to persist in recruitment within both academic and clinical spheres of U.S. medicine [10-12]. The Association of American Medical Colleges (AAMC) reports that advancements of women and URMs, particularly African Americans/Blacks, Hispanics, Asian/Pacific Islanders, and Native Americans in medical academic faculty, have not mirrored the proportional representation seen in their respective community demographics [7,13,14].

We conducted a literature search and found no study addressing the longitudinal trends in the diversity of microbiology faculty within U.S. medical schools. Our research aims to examine patterns in gender, race, and ethnicity across academic ranks and department chairs during the last 55 years. The goal is to comprehend the evolving demographics of microbiology, pinpoint areas for enhancement in diversity, and identify groups that might be particularly affected by the cessation of affirmative action.

## Materials and methods

In this retrospective study, the Institutional Review Board approval was not sought as the data used were publicly available and non-identifiable. The study population consisted of full-time microbiology faculty at schools in the United States, and the data spanned a time frame from 1967 to 2021 [15-17].

The data were distributed in 220 separate Excel sheets. Python coding is used to extract relevant data into dedicated Excel sheets. The faculty members were categorized into academic ranks: professor, associate professor, assistant professor, instructor, and leadership position of department chair. Also, faculty members were categorized based on tenure status, i.e., tenured, on-track, not on-track, and unavailable groups.

Additionally, the faculty members, tenure holders, and faculty chairs were classified into ten racial groups, namely American Indian or Alaskan Native, Asian, black or African American, Hispanic, Latino or of Spanish origin, Native Hawaiian or other Pacific Islander, white, other, multiple race Hispanic, multiple race non-Hispanic, and unknown. Furthermore, the faculty data were further stratified by gender, distinguishing between male and female faculty members and chairs.

To simplify the results, we combined data from the Native Hawaiian or other Pacific Islanders, other, multiple-race Hispanic, and multiple-race non-Hispanic groups into a single category labelled "other." The analysis depicted gender and racial distributions by year and across academic ranks, department chairs, and tenure status. Proportions, percentage changes of each gender, and racial category across each academic rank, department chair, and tenure status were calculated and averaged over the 55 years of this analysis.

The data were also fitted with a Poisson regression model to evaluate the annual percent change by gender and race within each faculty rank, tenure status, and chair. The 55-year data were grouped into five-year periods for analysis to enhance clarity. Data on microbiology students were obtained from the Association of American Medical Colleges, encompassing information on matriculants at U.S. medical schools categorized by gender, race, and ethnicity from 1967 to 2021 to compare trends in faculty members and microbiology matriculants [18]. Microsoft Excel (Microsoft Corporation, Redmond, Washington) and the open-source R Project for Statistical Computing (version 3.5.1, 2018, R Foundation for Statistical Computing, Vienna, Austria) were used for data analysis.

## Results

Microbiology faculty representation by race and gender

**Figure 1:**
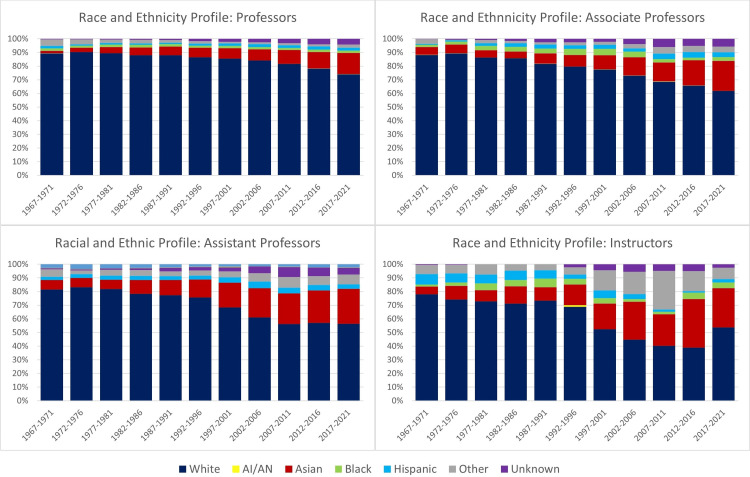
Annual percentage of full-time US microbiology faculty per race and ethnicity from 1967 to 2021 AI/AN = American Indian or Alaskan Native

The analysis of faculty rankings in the field of microbiology revealed a predominant presence of white faculty members throughout the study period from 1967 to 2021 (Figure 1). Except for instructors, the proportion of white faculty consistently exceeded 60% in each academic rank every five-year period. Notably, the representation of white faculty increased with higher academic ranks.

In contrast, the proportion of Asian faculty decreased as academic rank advanced during each five years of the study. For instance, in 2016-2021, white faculty constituted 53.7% of instructors, 57.7% of assistant professors, 61.8% of associate professors, and 74% of professors. During the same period, Asian faculty comprised 28.8% of instructors, 25.5% of assistant professors, 21.9% of associate professors, and 15.4% of professors. Asian faculty members are relatively well represented at all ranks except for the professorial level compared to their representation among matriculants in U.S. medical schools (20.9%).

**Figure 2:**
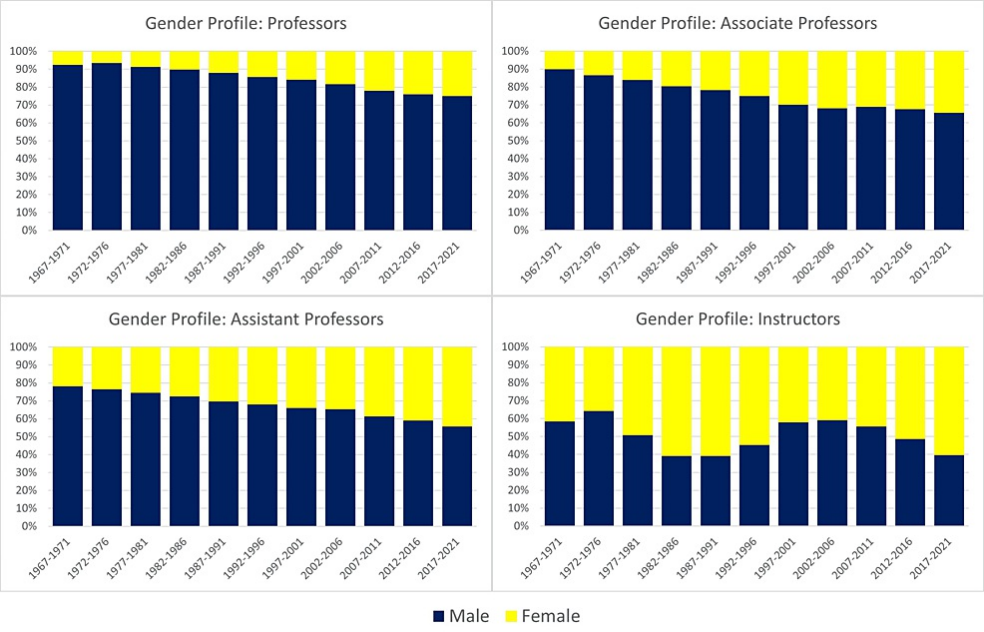
Annual percentage of full-time US microbiology faculty per gender from 1967 to 2021.

Women also had decreased representation as academic rank increased across each five-year span of the study (Figure 2). In 2017-2021, women comprised 60.4% of instructors, 44.3% of assistant professors, 32.2% of associate professors, and 25% of professors. Comparatively, women were underrepresented at higher academic ranks relative to their representation among matriculants in U.S. medical schools during the same period (34.7%).

The analysis of trends in the professorial rank showed that Asian and Hispanic microbiologists experienced the highest annual percentage increase of 31.4% and 17.2%, respectively, over the study period (Table 1). In the associate professor rank, Asian and Hispanic faculty increased by approximately 25%, whereas other racial and ethnic groups experienced growth rates of less than 10%. Among assistant professors, Asian faculty had the most significant increase of 28.9%, surpassing other groups, including black (5.3%), Hispanic (15.3%), and white (5.3%) faculty. In the instructor rank, faculty who identified as Asian experienced the most substantial increase at 26.2%.

**Table 1:** Average annual count and annual percent change in microbiology faculty per race and ethnicity from 1967 to 2021 The data is based on the Association of American Medical Colleges faculty roster.

	Average Annual Count (Percent Change)						
Faculty	American Indian or Alaska Native	Asian	Black or African American	Hispanic, Latino, or of Spanish Origin	White	Other	Unknown
Professors	2 (12.8)	270 (31.4)	55 (9.0)	56 (17.2)	2752 (7.6)	71 (46.3)	67 (0.0)
Associate Professors	1 (8.9)	237 (24.9)	67 (9.4)	59 (25.7)	1601 (3.9)	67 (44.2)	60 (22.3)
Assistant Professors	1 (0.0)	387 (28.9)	52 (5.3)	80 (15.3)	1546 (5.3)	87 (39.5)	122 (18.4)
Instructors	0 (0.0)	69 (26.2)	12 (8.8)	15 (-9.3)	203 (0.0)	9 (46.6)	42 (19.0)
Chairs	0 (0.0)	26 (31.9)	11 (-7.2)	17 (6.7)	473 (-2.9)	4 (12.2)	6 (-28.4)
Tenured	1 (0.0)	69 (2.9)	18 (0.2)	17 (0.5)	664 (10.5)	19 (0.0)	15 (0.0)
On-Track	0 (0.0)	41 (1.7)	7 (0.1)	8 (0.3)	237 (2.8)	14 (0.0)	15 (0.0)
Not On-Track	0 (0.0)	69 (2.6)	6 (0.2)	7 (0.1)	206 (4.9)	5 (0.2)	21 (0.8)
Unavailable	0 (0.0)	7 (0.2)	4 (0.1)	7 (0.0)	27 (0.2)	1 (0.0)	2 (0.0)

Regarding gender trends, female faculty saw an increase in the professor ranks by 26%, associate professor by 21.3%, assistant professor by 18.2%, and instructor by 8.5% during the study period (Table 2). In contrast, male faculty increased in the ranks of professor by 7%, associate professor by 4.4%, assistant professor by 6.8%, and instructor by 5.1%.

**Table 2:** Average annual count and annual percent change in microbiology faculty per race and ethnicity from 1967 to 2021 The data is based on the Association of American Medical Colleges faculty roster.

	Average Annual Count	
	(Percent Change)	
Faculty	Male	Female
Professors	2737 (7.0)	540 (26.0)
Associate Professors	1556 (4.4)	540 (21.3)
Assistant Professors	1506 (6.8)	773 (18.2)
Instructors	182 (5.1)	172 (8.5)
Chairs	466 (-4.1)	73 (23.3)
Tenured	652 (2.2)	153 (5.4)
On-Track	232 (1.1)	91 (3.4)
Not On-Track	190 (4.4)	132 (5.5)
Unavailable	32 (1.0)	16 (5.9)

Microbiology chairs by race and gender

**Figure 3:**
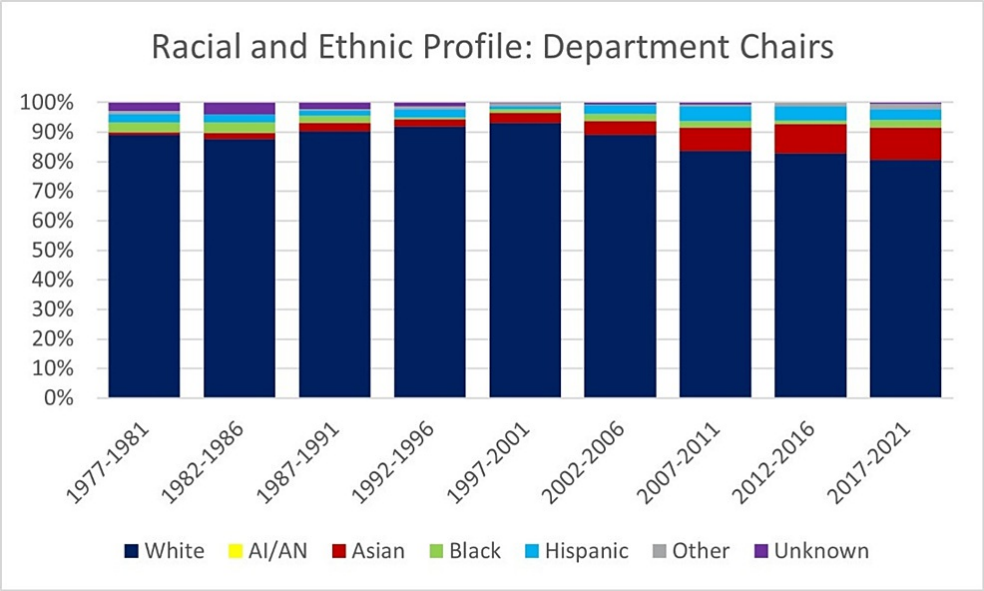
Annual percentage of microbiology department chairs per race and ethnicity from 1967 to 2021 AI/AN = American Indian or Alaskan Native

Throughout the study period, white faculty members consistently held the majority of microbiology department chair positions every five-year period (Figure 3). White chairs represented 89.4% of all chairs in 1966-1971, gradually decreasing to 80.2% in 2016-2021. In contrast, chairs of Asian, black, and Hispanic backgrounds collectively represented less than 20% of the total chairs in each block, with no representation of American Indian chairs during the study period. Over the 55-year period, the percentage of Asian individuals experienced the most robust surge, with a rate of 31.9% per year. Meanwhile, Black chairs showed the sharpest decline, with a rate of 7.2% decrease per year (Table 1).

**Figure 4:**
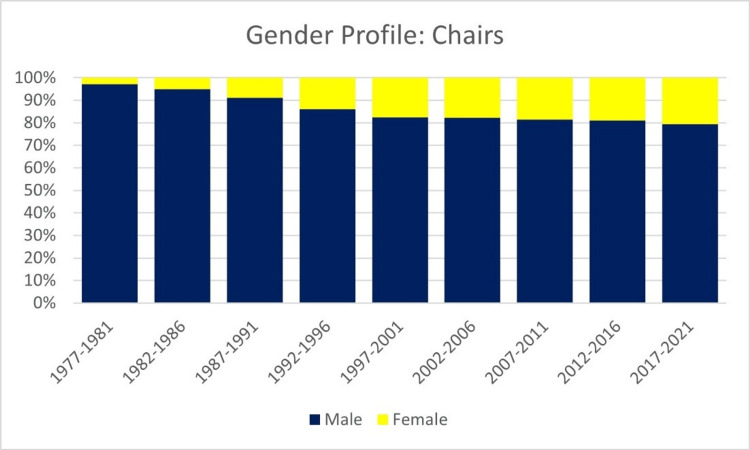
Annual percentage of microbiology department chairs per gender from 1967 to 2021

The proportion of women holding microbiology department chair positions increased from 2.9% in 1966-1971 to 20.5% in 2016-2021 (Figure 4). Over the study period, the number of women department chairs increased by 23.3% annually, while the number of male chairs decreased by 4.1% annually (Table 2).

Microbiology tenure positions by race and gender

**Figure 5:**
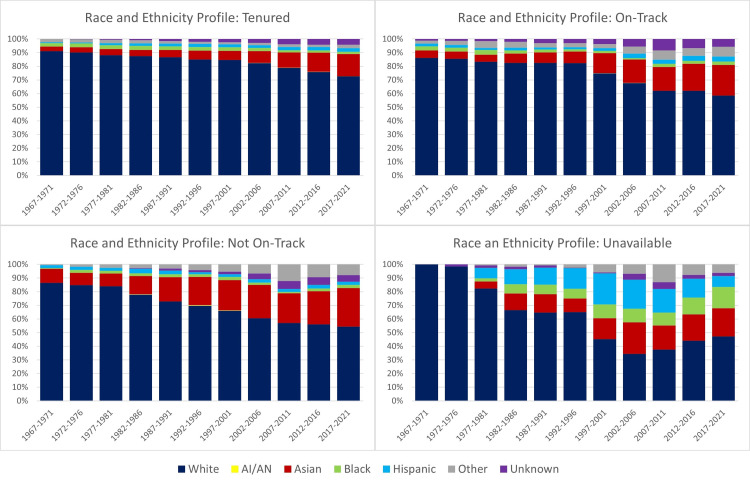
Annual percentage of microbiology tenure tracks per race and ethnicity from 1967 to 2021 AI/AN = American Indian or Alaskan Native

The overall distribution of academic microbiology faculty members in terms of tenure positions revealed that 49.4% were tenured, 19.8% were on track for tenure, and 19.8% were not on track, with 11% unavailable. White individuals comprised the largest proportion of tenured faculty (86.5%), followed by Asian (8.9%), black (2.3%), Hispanic (2.0%), and American Indian (0.0%) individuals. A similar trend was observed for faculty members on track and not on track, with an increasing representation of Asian individuals (Figure 5).

Over the 55 years, the percentage of white tenure holders decreased from 88.9% in 1967-1971 to 68% in 2017-2021, while Asian tenure holders increased from 3.2% to 15.3% during the same period. The percentages of black, Hispanic, and American Indian tenure holders experienced less than 2% fluctuations throughout the study period (Table 1). By 2017-2021, the racial profile of tenure holders more closely reflected the racial profile of individuals matriculating to U.S. microbiology schools.

**Figure 6:**
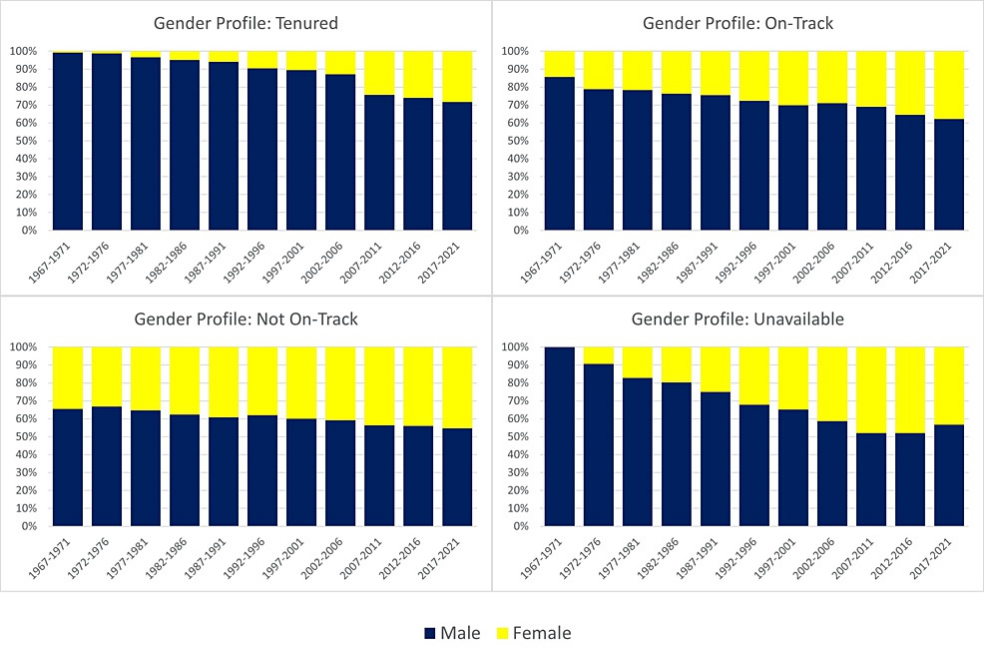
Annual percentage of microbiology tenure tracks per gender from 1967 to 2021.

While males held a majority of tenure, on-track, and not on-track positions during the study period (Figure 6), there was a notable positive trend in the percentage of female tenure holders from 0.7% in 1967-1971 to 28.3% in 2017-2021, with an annual percent change of 5.4% (Table 2). Nevertheless, males dominated tenure positions at the end of the study period, holding 71.6% of all tenure positions. Similar trends were observed for on-track positions (Figure 6).

## Discussion

The findings of our study reveal encouraging trends for women and underrepresented minorities (URM) among microbiology faculty, particularly for Asian representation. However, the situation for faculty of other races and ethnicities appears less promising.

Throughout the study period, there was a noticeable enhancement in the presence of Asian faculty and leaders in the field of microbiology. Despite this progress, disparities persist for black, Hispanic, and Native American microbiologists in both faculty and chair positions. Interestingly, the proportions of these underrepresented minority (URM) groups within microbiology faculty mirror their representation among matriculants. For instance, in the 2017-2021 period, black, Hispanic, and Native American groups constituted 1.7%, 2.2%, and 0.2% of microbiology matriculants, indicating broader challenges and disparities that hinder minority access to higher education. The cessation of affirmative action may exacerbate these challenges, compounding the difficulties these groups face.

By taking a closer look at the individual minority groups, our data also showed discrepancies in the proportion of academic positions held by individuals from each group. We found that Asian faculty had the most significant increase in representation over the past 55 years, followed by Hispanic and Black. Comparing this to U.S. census data spanning from 1970 to 2020, there were significant population increases: Asian population by 1500%, Blacks by 1800%, Hispanics by 717%, and Native Americans by 1223% [19-23]. In the same period, the White population increased by 129% [21]. Analyzing our data, we observed an approximately 38-fold increase in Asian microbiology faculty over the past 55 years, potentially influenced by the surge in the Asian population in the U.S. However, this trend was not mirrored in other underrepresented populations. Despite substantial growth in Black and Hispanic microbiology, faculty representation only saw about a four-fold increase. This points to potential disparities in mentioned minority groups in terms of accessing higher education [24].

The positive trend observed in the annual increase of women in microbiology faculty positions, academic ranks, chairs, and tenure positions may be attributed to deliberate efforts to enhance diversity within the discipline. Initiatives such as the ACR Commission for Women and General Diversity have likely played a role and can serve as models to be replicated or supplemented at the institutional level. By strengthening interest, recruitment, and retention in microbiology throughout the science education pipeline, we can continue to build on this progress.

While women are proportionally represented among microbiology faculty compared to their matriculant counterparts, the diminishing proportion of women as academic ranks increase over the 55-year study period indicates attrition from academics or a lack of promotion opportunities for women. A dedicated longitudinal study including promotion-related factors is recommended to understand the primary factors contributing to this consistent trend. Our findings underscore previous research showing that gender and racial bias influence various aspects of academia and professional life, such as grading, hiring, mentoring, tenure, promotion, respect, grant proposal success, and pay [25-35].

Numerous factors have been proposed to explain these disparities, including biases in training and hiring practices, the impact of family responsibilities on career trajectories, inadequate support for primary caregivers, perceived competency differences, and variations in research productivity as measured by publications [36-40]. These factors often accumulate, leading to disparities for both individuals and the broader scientific community [40-42]. Nonetheless, this trend highlights the greatest group at risk as a result of the end of affirmative action, with African-American, Hispanic, and American Indian minority groups likely to be disproportionately affected by their already low representation among microbiology faculty.

One persistent issue that remains to be solved is the need for more women and URMs in decision-making roles within academia, industry, and science policymaking. Despite efforts to enhance recruitment and retention of women and URMs in science, the percentage of women decreases in more senior academic ranks, leading to what is commonly referred to as the sticky floor, broken ladder and glass ceiling; another name for this phenomenon is 'leaky pipeline' [43-45].

Moreover, the findings of this study might suggest that affirmative action was not a universally effective strategy for certain populations, such as African Americans, Hispanics, and Native Americans. It is crucial to acknowledge that affirmative action was already prohibited in specific states prior to the recent ruling, including California in 1996 and Florida in 1999 [46,47]. This trend extends beyond California, with studies revealing that Florida, Washington, Texas, Michigan, and Nebraska banned affirmative action and, as a result, had decreasing diversity, even at the medical school level [47,48]. However, what raises concern are the potential implications of the recent Supreme Court ruling, which may lead to a decline in diversity similar to what has been observed in individual states.

Addressing these issues necessitates a comprehensive approach by institutions and scientific community members. Measures such as creating diverse selection committees, adhering to transparent, objective criteria in recruitment and promotions, implementing family-supportive laws, and addressing gender-specific needs can help retain more women microbiologists and facilitate their advancement [49]. Additionally, holding leaders accountable for promoting diversity among microbiology students can be a formal motivation to increase diversity within the field. By collectively taking action, we can strive toward a more equitable and inclusive future for microbiology and the scientific community as a whole.

Limitations

Our study has its share of limitations, including the inherent constraints with the data and collection process. Specifically, the categorization of communities into broader racial groups, like grouping Chinese, Vietnamese, and Filipino populations under "Asian," may result in an incomplete depiction of factors contributing to under-representation in individual communities. Additionally, the binary classification of sex based on identified gender may not fully capture the self-identification of a subset of internists. The research leans on publicly accessible and non-identifiable data, potentially constraining the analysis's depth and specificity. This approach might miss out on some data intricacies and nuances. Future investigations might benefit from incorporating surveys or interviews to overcome the limitations tied to public data, delving deeper into faculty members' perspectives. It's essential to note that the study focuses solely on faculty demographics, neglecting crucial aspects like career advancement, publication output, and funding achievements. A more holistic grasp of diversity in microbiology could emerge by exploring these additional factors.

Moreover, the voluntary and self-reported nature of the faculty roster data means that information from faculty who choose not to participate is not included, introducing a potential bias. The data is aggregated for microbiology faculty across all U.S. medical schools, preventing an analysis of demographic trends in smaller subsets, such as by region or state, which could offer insights into regional disparities. Given the diverse population demographics across U.S. states, further studies could enhance understanding by adapting and subcategorizing the data by state, exploring whether diversity among microbiology faculty varies across different regions. Likewise, it's important to note that this study focused solely on race and sex, excluding other protected population factors crucial for diversity, such as religion, sexual orientation, gender identification, disability, socioeconomic status, and rural living. Ultimately, this study has purely focused on the microbiology faculty members, limiting its generalizability to the whole academia. Including vast data in future studies can alleviate this limitation.

## Conclusions

In conclusion, our research provides a valuable baseline for future evaluation and improvements. Our study reveals progress and challenges in increasing gender and racial diversity within microbiology. While there have been positive improvements in the representation of Asian faculty and women, disparities persist for other underrepresented minority groups. Yet, the termination of affirmative action in the U.S. puts all of this at risk, and people from African-American, American Indian, and Hispanic backgrounds are expected to bear the brunt of the consequences. These findings underscore the need for comprehensive efforts to address biases in recruitment, promotion, and decision-making roles, aiming to create a more equitable and inclusive scientific community. Ultimately, it is essential to continue working to enhance diversity, equity, and inclusion in microbiology, fostering a more innovative and equal scientific environment for all.
